# Durable complete response of advanced hepatocellular carcinoma using cannabis oil: a report of two cases

**DOI:** 10.1186/s42238-025-00353-0

**Published:** 2025-11-24

**Authors:** Pieter F. van den Berg, Frans van der Heide, Simeon J.S. Ruiter, Jules J.G. Slangen, Derk Jan A. de Groot, Evert van den Broek, Frederik J.H. Hoogwater, Maarten W. Nijkamp

**Affiliations:** 1 Department of Hepato-Pancreato-Biliary Surgery and Liver Transplantation, University Medical Center Groningen, Groningen, The Netherlands; 2 Department of Gastroenterology and Hepatology, University Medical Center Groningen, Groningen, The Netherlands; 3 Department of Radiology, University Medical Center Groningen, Groningen, The Netherlands; 4 Department of Medical Oncology, University Medical Center Groningen, Groningen, The Netherlands; 5 Department of Pathology, University Medical Center Groningen, Groningen, The Netherlands

**Keywords:** Hepatocellular carcinoma, Cannabinoid, Cannabis, Tumor response, Treatment

## Abstract

**Background:**

Hepatocellular carcinoma (HCC) is a leading cause of cancer-related mortality worldwide with a grim prognosis. Current treatment options for advanced HCC are limited, and a large proportion of patients is not amenable to any form of treatment, with best supportive care as the only remaining option. Meanwhile, the use of cannabis-derived products is rising in oncological patients who are seeking symptom relief. Cannabinoids, similar to endogenous endocannabinoids, have shown promise in recent preclinical cancer research due to their ability to interact with various signaling pathways and molecular mechanisms of interest.

**Case presentation:**

In this report, we present two patients (A aged 82 and B 77, respectively) with advanced HCC with a high tumor burden who demonstrated durable and complete regression after use of cannabis oil (A 10% delta-9-tetrahydrocannabinol (THC) and 5% cannabidiol (CBD), two droplets sublingually three times daily and B 15% THC and 2% CBD, 5 droplets sublingually two times daily) for symptom relief. The observations in this report build on previous (pre)clinical research highlighting the potential anti-tumor qualities of cannabinoids and stress the need for clinical trials investigating the anti-tumor effects of cannabinoids in cancer patients.

**Conclusion:**

Based on the two cases presented here, we call for further research into the potential beneficial effect of cannabinoids in patients with advanced HCC.

**Supplementary Information:**

The online version contains supplementary material available at 10.1186/s42238-025-00353-0.

## Background

Hepatocellular carcinoma (HCC) is currently the sixth most common neoplasm globally and the third most frequent cause of cancer-related death in the world. HCC frequently occurs in patients with chronic liver disease, most often in the presence of liver cirrhosis. The development of HCC in a cirrhotic liver is a multistep process that involves sustained inflammatory damage, including hepatocyte necrosis and regeneration, associated with fibrotic deposition in the liver. Most patients present with an advanced stage of HCC, as early stages often develop asymptomatic (Forner et al. 2018). Despite recent developments regarding systemic treatment with atezolizumab/bevacizumab, up to one third of the patients presenting with advanced HCC are unsuitable for any form of treatment (Meer et al. 2016; Costentin et al. 2018). Indeed, patients with advanced HCC form a complex clinical challenge for treatment due to their compromised hepatic function associated with chronic liver disease and their reduced drug tolerance as a result. For these patients, only best supportive care with symptom relief remains as a last resort. The prognosis of these patients is poor, with an overall survival of approximately eight months (Forner et al. 2018). 

Nowadays, an increasing number of oncological patients use cannabis-derived products for symptom relief (Oelen et al. 2023; Woerdenbag et al. 2023). The role for cannabinoids in the palliative setting is known for symptomatic applications such as the prevention of chemotherapy-induced nausea, pain control, appetite stimulation and reducing anxiety as presented in recent systematic reviews highlighting the potential anti-cancer activity of cannabinoids across various tumor types, encompassing gastrointestinal, lung, breast and prostate cancers (Woerdenbag et al. 2023; Shah et al. 2023). Here, we report two cases of durable complete regression of advanced HCC after using cannabis oil for symptom relief.

## Case presentation

### Patient A

**Fig. 1:**
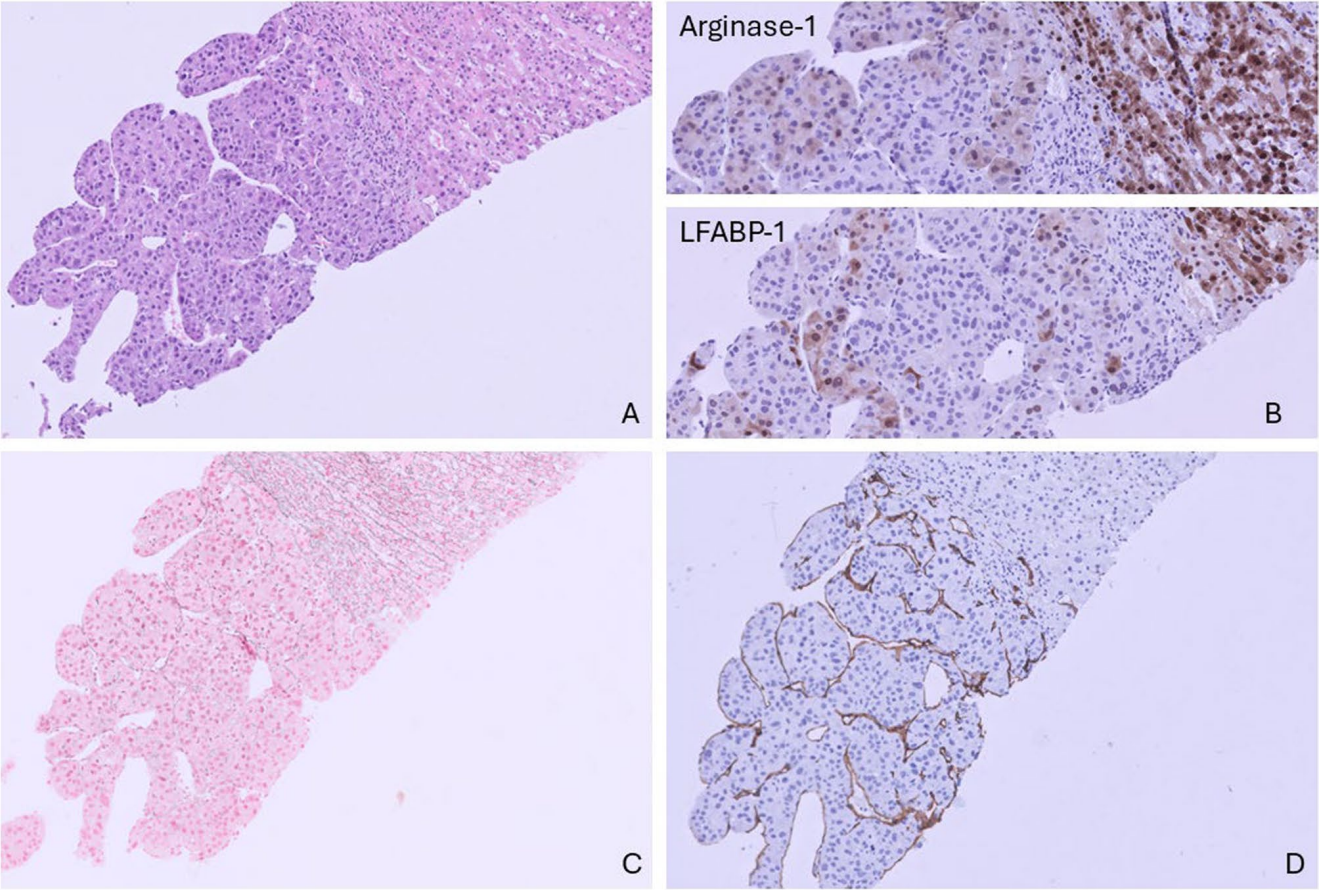
Liver biopsy showing a moderately differentiated hepatocellular carcinoma in a noncirrhotic background. Standard HE staining, 20X magnification (**a**). Variable cytoplasmic and nuclear positivity of Arginase-1 and LFABP-1 in the tumor cells (**b**). Gomori’s silver stain showing fragmentation and loss of the reticuline network (**c**). Diffuse capillarization of sinusoids confirmed by CD34 stain (**d**)

The first patient, an 82-year-old male, was referred to our hospital for the treatment of a solitary liver tumor. He was transferred from another hospital where he was analyzed for abdominal pain and weight loss. Ultrasound revealed a large tumor in the right lobe of the liver. The serum level of alpha-fetoprotein (AFP) was elevated; 59 µg/L. Magnetic resonance imaging (MRI) demonstrated a tumor of 10 cm in diameter in liver segments 5, 7 and 8. There was no history of excessive alcohol intake. Screening blood tests for causal factors of HCC, including hepatitis B and C, were negative. Because of inconclusive imaging results, a histologic biopsy was taken and demonstrated a moderately differentiated hepatocellular carcinoma in a noncirrhotic background (Fig. 1). The tumor had a trabecular growth pattern and comprised of cells with enlarged nuclei with multiple mitotic figures. Hepatocellular origin was further supported by variable cytoplasmic and nuclear expression of Arginase-1 and LFABP-1 (Fig. 1b). The tumor cells were Keratin 18 positive. A canalicular pattern was seen by poly CEA immunohistochemistry. BerEp-4, Keratin 7, 19 and 20 were all negative by additional examination (not shown). Gomori’s silver stain confirmed loss and fragmentation of reticuline fibers (Fig. 1c). The present capillarization of sinusoids was confirmed by CD34 (Fig. 1d).

The patient refused extensive surgical resection and declined palliative systemic therapy for HCC. To reduce his abdominal complaints, he started using cannabis oil shortly after his diagnosis. The cannabis oil was obtained via an unknown online supplier and the product label stated the oil contained 10% delta-9-tetrahydrocannabinol (THC) and 5% cannabidiol (CBD). He did not experience any side effects using two droplets sublingually three times daily.

**Fig. 2:**
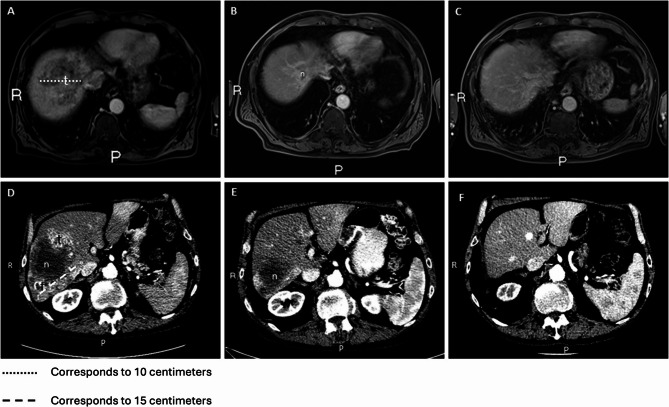
Contrast-enhanced magnetic resonance (MR) images of an 82-year-old patient with advanced HCC (**a**) at diagnosis (**b**) at 6-month follow-up after using cannabis oil (**c**) at 6 years after diagnosis. Contrast-enhanced computed tomography (CT) images of a 77-year-old patient with advanced HCC (**d**) at diagnosis (**e**) at 6-month follow-up after using cannabis oil (**f**) at almost 4 years follow-up after using cannabis oil. N necrosis, t tumor

Although the patient was sent to the general practitioner for best supportive care after his diagnosis, he was readmitted for oncological follow-up. At this first follow-up after 6 months using the cannabis oil, his abdominal complaints had resolved, and AFP levels were normalized to 2 µg/L. MRI demonstrated regression of the tumor to a size of 5.1 cm. The patient continued the use of cannabis oil, and the tumor continued reducing in size. Approximately two years after the diagnosis, the tumor was undetectable on MRI (Fig. 2A-C). Until today, almost 8 years after diagnosis, the tumor has not been detected again on imaging studies and AFP levels have remained normal.

### Patient B

The second patient, a 77-year-old male was referred to our hospital with undesired weight loss, a liver mass detected on ultrasound, and an AFP of 40,950 µg/L. He had a history of alcohol abuse. Screening blood tests for other causal factors of HCC, including hepatitis B and C, were negative. Computed tomography (CT) studies demonstrated a large tumor of 15.6 cm with central necrosis in liver segments 6, 7 and 8. A second lesion of 2.5 cm was located in segment 5. HCC was diagnosed based on typical imaging characteristics (arterial enhancement with wash-out in the late phase) combined with highly elevated levels of AFP and des-gamma carboxyprothrombin (22,142 AU/L) (Forner et al. 2018). Both tumors were deemed unresectable and the patient refused palliative treatment options for HCC, including selective internal radiotherapy (SIRT). In order to improve appetite and gain weight, he started to use cannabis oil upon diagnosis. The cannabis oil was obtained via an unknown online supplier and the product label stated the oil contained 15% THC and 2% CBD. He did not experience any side effects using 5 droplets sublingually two times daily.

After 3 months using cannabis oil, his clinical condition had improved, and he had gained weight. Upon imaging, the tumors had reduced in size from 15.6 to 9.2 cm in diameter and 2.5 to 1.9 cm, respectively. Afterwards, he continued to use cannabis oil and approximately 15 months after diagnosis the AFP had normalized to 2 µg/L. On CT, no vital tumor tissue was visible anymore, only rest necrosis (Fig. 2D-F). To this day, almost 5 years after diagnosis, imaging studies do not demonstrate any recurrent disease and AFP levels are normal.

In both patients, no significant dietary, lifestyle or other supportive interventions were initiated during the follow-up period aside from the reported use of cannabis oil. Neither patient had a history of recreational or medical cannabis use, nor of other cannabis-related substances, prior to their cancer diagnosis. Although both patients reported no adverse effects, systematic adverse event monitoring was not performed.

## Discussion

In this report we present two cases demonstrating a durable and complete regression of advanced HCC, associated with the use of cannabis oil. Although spontaneous regression of hepatocellular carcinoma occurs in approximately 0.4% of cases, it is frequently temporary (Oquiñena et al. 2009). Nevertheless, while the temporal association is compelling, causality cannot be inferred. These cases should therefore be regarded as hypothesis-generating rather than conclusive.

Numerous studies have been performed on the biological activities of cannabinoids. Research has mainly focused on THC and CBD. THC is the primary source of the psychoactive side effects of cannabis, in contrast to CBD which has anti-inflammatory and anxiolytic properties (Guzmán 2003; Hinz and Ramer 2019). Next to the palliative effects, it has been suggested that cannabinoids may have an anti-tumor effect as well. Both preclinical in vitro and in vivo studies have demonstrated that cannabinoids (including THC and CBD) elicit effects at different levels of cancer progression, including inhibition of proliferation, neovascularization, invasion and chemoresistance, induction of apoptosis as well as enhancement of tumor immune surveillance (Guzmán 2003; Hinz and Ramer 2019). A recent review by Shah et al. summarized the anti-tumor effects of cannabinoids in several malignancies, including their roles in suppressing angiogenesis, promoting apoptosis, and modulating tumor immunity across brain, breast, and gastrointestinal cancers (Shah et al. 2023). These mechanisms may similarly apply to HCC where indeed, cannabinoids can inhibit HCC tumor cells via apoptosis induction, proliferation inhibition and reduction of invasion and migration (Rao et al. 2019; Xu et al. 2015; Vara et al. 2011, 2013; Pellerito et al. 2010). Esmaeli and Dehghanpour Dehabadi recently highlighted the therapeutic potential of CBD in HCC by outlining several mechanisms of action including apoptosis, suppression of epithelial-mesenchymal transition (EMT), inhibition of the PI3K/AKT/mTOR pathway and enhancement of chemotherapy efficacy (Esmaeli and Dehghanpour Dehabadi 2025). Although the exact mechanisms remain to be elucidated, the role of uncontrolled autophagy leading to cell death of tumor cells both in vitro and in vivo may play an important role (Vara et al. 2011, 2013). 

Several clinical studies investigated cannabinoid levels and receptor status in HCC patients. Yang et al. compared HCC tissue samples from 67 HCC patients with adjacent non-tumorous tissue. They demonstrated that the expression of the endocannabinoids anandamide (AEA) and 2-arachidonylglycerol (2-AG) were significantly different between tumor tissue and normal liver tissue. Also, the expression of cannabinoid-binding (CB) receptors CB1 and CB2 were different between tumor tissue and normal liver tissue (Yang et al. 2019). Moreover, Xu et al. demonstrated that the overexpression of both CB1 and CB2 receptor in tumor tissue was associated with improved overall survival in 64 HCC patients (Xu et al. 2006). 

Notably, the two patients used formulations with different THC/CBD ratios (10:5 versus 15:2), potentially influencing clinical response. While THC is often linked to pro-apoptotic and anti-proliferative effects, CBD has been shown to exert anti-inflammatory, anxiolytic and complementary anti-tumor activities. The interplay between these cannabinoids may affect efficacy, warranting further pharmacodynamic studies. Importantly, no pharmacokinetic measurements or serum cannabinoid levels were available for the presented cases, and the formulations used were not standardized in terms of bioavailability. This limits reproducibility and underscores the need for future studies to incorporate such data.

While these cases suggest a possible association between cannabis oil use and tumor regression in advanced HCC, the observational nature of this report precludes causal inference. Furthermore, the cannabis oil used was not GMP-certified, and no structured adverse event monitoring was undertaken, which limits safety assessment and generalizability. Additionally, the exact suppliers of the cannabis oil in these cases are unknown and the exact cannabinoid content of the cannabis oil was not verified by our laboratory, as labeled and observed cannabinoid content may differ (Hazekamp 2018). While preclinical data consistently support cannabinoid-mediated anti-tumor effects, clinical evidence remains limited. The recently initiated CanHep study (EUDRACT 2018–004505-34; NCT06518434) will prospectively evaluate the efficacy of cannabis oil in advanced HCC. In this pilot study, 20 untreatable patients with advanced HCC will be treated with cannabis oil (THC 10% and CBD 5%) for a duration of 9 months. Compared to our anecdotal observations, this study includes systematic patient-tailored dosing, monitoring and mechanistic analyses, which will be critical for validating the hypothesis generated by these case reports. Nonetheless, these findings highlight the urgency of conducting controlled clinical trials to evaluate the therapeutic potential and underlying mechanisms of cannabinoids in HCC, such as the CanHep study. Exploring the effect of medicinal cannabis oil in HCC patients may result in a new field of research including the search for a possible working mechanism using patient-derived tumor slices and tumoroids.

## Conclusion

The authors present two cases of durable and complete remission in two patients with advanced hepatocellular carcinoma using cannabinoids, thus stressing the call for further research into the anti-tumor effects of cannabinoids in this patient population with limited therapeutic options. These findings are hypothesis-generating and underscore the urgent need for controlled clinical trials.

## Supplementary Information

Supplementary Material 1.

## Data Availability

Data sharing is not applicable to this article as no datasets were generated or analyzed during the current study.

## Abbreviations

AFP: Alfa fetal protein
CBD: Cannabidiol
CT: Computed tomography
HCC: Hepatocellular carcinoma
MRI: Magnetic resonance imaging
SIRT: Selective internal radiotherapy
THC: Delta-9-tetrahydrocannabinol
